# Dairy-Gut Microbiome Interactions: Implications for Immunity, Adverse Reactions to Food, Physical Performance and Cardiometabolic Health—A Narrative Review

**DOI:** 10.3390/nu17203312

**Published:** 2025-10-21

**Authors:** Javier Modrego, Lisset Pantoja-Arévalo, Dulcenombre Gómez-Garre, Eva Gesteiro, Marcela González-Gross

**Affiliations:** 1 ImFINE Research Group, Department of Health and Human Performance, Universidad Politécnica de Madrid, 28040 Madrid, Spain; javier.modrego@upm.es (J.M.); l.pantoja@upm.es (L.P.-A.); eva.gesteiro@upm.es (E.G.); marcela.gonzalez.gross@upm.es (M.G.-G.); 2 Instituto de Investigación Sanitaria Hospital Clínico San Carlos (IdISSC), 28040 Madrid, Spain; 3 Vascular Biology, Microbiota Laboratory, Hospital Clínico San Carlos, Department of Physiology, School of Medicine, Universidad Complutense de Madrid (UCM), IdISSC, 28040 Madrid, Spain; mgomezgarre@salud.madrid.org; 4 Biomedical Research Networking Center in Cardiovascular Diseases (CIBERCV), Carlos III Health Institute, 28029 Madrid, Spain; 5 Biomedical Research Networking Center of Pathophysiology of Obesity and Nutrition (CIBERobn), Carlos III Health Institute, 28029 Madrid, Spain

**Keywords:** cardiometabolic risk factors, dairy products, food hypersensitivity, functional food, gut microbiome, physical functional performance, metagenomics

## Abstract

**Background/Objective:** Milk and fermented dairy products are widely consumed functional foods and beverages, offering not only essential nutrients but also bioactive compounds with potential to modulate host immunity, metabolism, and the gut microbiome. This narrative review aims to synthesize current knowledge on the relationship between dairy consumption, gut microbiome, immune modulation, adverse reactions to food, physical performance and cardiometabolic health. **Methods:** An extensive literature analysis was conducted to explore how milk and fermented dairy products modulate the gut microbiome and influence the immune and cardiometabolic health. This study synthesis focused on key dairy bioactive compounds, such as probiotics, miRNAs, milk-derived peptides and exosomes and on evaluating their proposed mechanisms of action in inflammation and metabolic regulation, and their possible influence on physical performance through gut–microbiome interactions. Additionally, advances in metagenomic and metabolomic technologies were reviewed for their potential to uncover host–microbiota interactions relevant to precision nutrition strategies. **Results:** Fermented dairy products have shown potential in promoting beneficial bacteria growth such as *Lactobacillus* and *Bifidobacterium*, short-chain fatty acid synthesis and reduction in proinflammatory biomarkers. Specific dairy-derived peptides and exosomal components may further support gut barrier integrity, immune regulation and improve physical performance and reduce cardiometabolic risk factors. Additionally, emerging evidence links individual gut microbiota profiles to specific metabolic responses, including tolerance to lactose and bovine milk proteins. **Conclusions:** Integrating microbiome science with traditional nutritional paradigms enhances our understanding of how dairy influences immune and cardiometabolic health. Overall, current evidence suggests that investigating dairy-microbiome interactions, alongside lifestyle factors such as physical activity, may inform future personalized nutrition strategies aimed at supporting metabolic and immune health.

## 1. Introduction

The health-promoting effects of fermented dairy products have been acknowledged since antiquity. Long before the advent of microbiology, ancient civilizations developed empirical preservation techniques such as inoculating fresh milk with small amounts of sour milk that not only extended shelf life but also inadvertently fostered the intake of beneficial microorganisms [1].

Scientific interest in these traditional practices has been substantially growing since the early 20th century, when Nobel laureate Professor Elie Metchnikoff proposed a groundbreaking theory about the health benefits of fermented milk products. Metchnikoff stated that autointoxication from putrefactive bacteria in the colon contributed to aging and that *Lactobacillus bulgaricus* in fermented milk products could counteract this effect. Thus, he hypothesized that lactic acid bacteria in fermented milk products could suppress harmful intestinal microbes and delay aging by observing the notable longevity of Balkan populations who consumed sour milk products regularly, thereby supporting gastrointestinal and systemic health [2].

This early hypothesis has since evolved into a comprehensive scientific framework, in which the gut microbiota is recognized as a central regulator of host physiology influencing conditions like obesity, diabetes, and inflammatory diseases [3,4]. Dairy products, especially fermented varieties, are now classified as functional foods due to their bioactive components such as γ-aminobutyric acid (GABA), conjugated linoleic acid (CLA), lactoferrin, lactoferricin, exopolysaccharides, short-chain fatty acids (SCFAs) and polyphenols (e.g., epigallocatechin gallate and chlorogenic acid), which exert immunological and cardiometabolic benefits beyond basic nutritional value [5,6]. These beneficial effects are thought to be mediated in part through the modulation of the gut microbiota, including increases in beneficial genera such as *Lactobacillus* and *Bifidobacterium* [7,8]. Importantly, recent evidence suggests that the dairy-gut microbiota interaction may also play a role in enhancing physical performance, potentially through mechanisms involving improved metabolic efficiency, reduced inflammation, and optimized nutrient absorption [9,10].

In this context, the present narrative review aims to synthesize the current knowledge on the functional constituents of milk and its fermented derivatives whose consumption is widespread globally. In this regard, we focus on their capacity to influence the gut microbiome and support immunity, tolerance to adverse reactions to food (ARF), improving physical performance and cardiometabolic health. Moreover, the emerging role of metagenomic technologies is discussed, offering novel insights for the development of precision nutrition strategies based on individual microbiome profiles.

## 2. Materials and Methods

**Figure 1:**
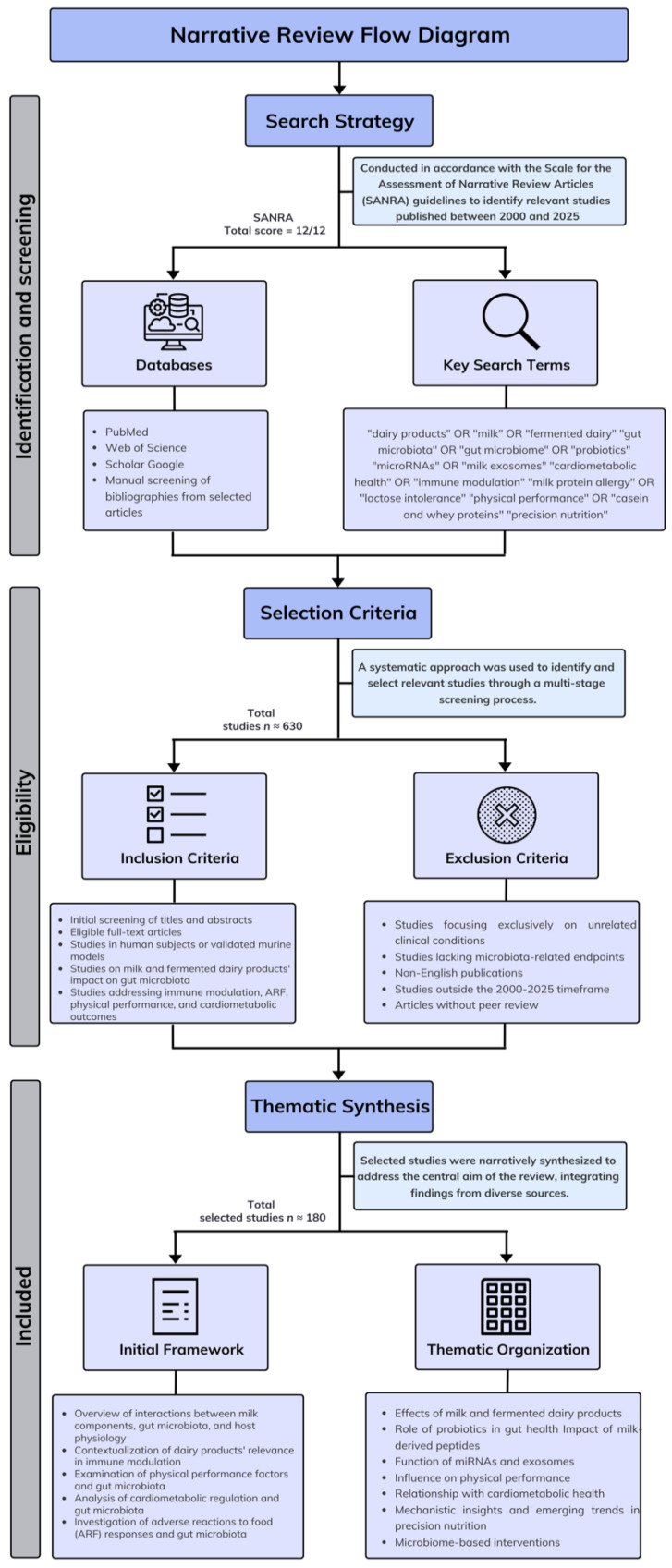
Flow diagram of the Narrative Review of Dairy-gut microbiome interactions: implications for immunity, adverse reactions to food, physical performance and cardiometabolic health. Own elaboration using Venngage Multimedia and Design Software version Beta 2025 (Venngage Inc., Toronto, ON, Canada).

A flow diagram detailing the narrative review process utilized in this study is schematically represented in Figure 1.

### 2.1. Search Strategy

This narrative review was conducted in accordance with the Scale for the Assessment of Narrative Review Articles (SANRA) guidelines [11]. A comprehensive literature search was performed using PubMed, Web of Science, and Google Scholar databases to identify relevant studies published between 2000 and 2025 (date range of eligible publications: 1 January 2003 to 31 July 2025). A preliminary search considering related keywords took place using the following Boolean equation: “dairy products” OR “milk” OR “fermented dairy” AND “gut microbiota” OR “gut microbiome” OR “probiotics” AND “microRNAs” OR “milk exosomes” AND “cardiometabolic health” OR “immune modulation” OR “milk protein allergy” OR “lactose intolerance” OR “physical performance” OR “casein and whey proteins” AND “precision nutrition”. Eligible studies were selected based on five criteria: (1) relevance to probiotics, prebiotics, symbiotic, or fermented dairy; (2) focus on gut microbiota and its impact on immune, cardiometabolic, ARF or food sensitivities physical performance and overall health; (3) publication in peer-reviewed journals between 1 January 2003 and 31 July 2025; (4) availability in English or Spanish language; and (5) alignment with the predefined Boolean search strategy.

Additional references of interest were retrieved through personalized manual screening of bibliographies from selected articles. Studies involving either human subjects or biologically pertinent murine models were favored. The selection prioritized peer-reviewed articles that addressed the interplay between dairy intake, gut microbiome modulation, and health outcomes related to immunity, food sensitivities, physical performance and cardiometabolic risk. Potential sources of bias in this narrative review were minimized through a structured search strategy, transparent inclusion criteria, and critical appraisal of the literature. To reduce interpretive bias, endpoint data were contextualized with mechanistic insights and regulatory perspectives, and conflicting findings were acknowledged where applicable. This review favors English and Spanish research, potentially underrepresenting data from Asia, Africa, or non-Western dietary contexts.

### 2.2. Study Selection Process

Initial screening was performed by evaluating the titles and abstracts, followed by the assessment of eligible articles. Studies involving human subjects or validated murine models were prioritized, particularly those addressing the impact of milk and fermented dairy products on gut microbiota composition, immune modulation, ARF, physical performance and cardiometabolic outcomes. Articles focusing exclusively on unrelated clinical conditions or lacking microbiota-related endpoints were excluded from detailed analysis but occasionally referenced for background context. Discordant findings were managed through structured triangulation and contextual appraisal. Studies presenting conflicting results, particularly regarding the effects of probiotics, milk-derived exosomes, or microRNAs on immune or cardiometabolic outcomes, were not excluded but critically examined for differences in population characteristics, intervention design, dosage, and endpoint definitions. Following PubMed-indexed best practices (e.g., [12,13]), discordant data were analyzed and examined by study type (e.g., observational vs. interventional). Where inconsistencies persisted, findings were interpreted in light of biological plausibility, regulatory consensus (e.g., EFSA, FAO), and mechanistic coherence.

### 2.3. Data Synthesis

The selected studies were narratively synthesized, integrating findings from diverse sources to address the central aim of this review. An initial overview of the interactions between milk components, gut microbiota, and host physiology was included to contextualize the relevance of dairy products in immune modulation, physical performance, cardiometabolic regulation, and ARF responses. Key topics, such as the effects of milk and fermented dairy products, probiotics, milk-derived peptides, miRNAs, exosomes, physical performance or cardiometabolic health, were extracted and used to structure several thematic subsections. This approach facilitated the organization and interpretation of heterogeneous evidence, highlighting mechanistic insights and emerging trends relevant to precision nutrition and microbiome-based interventions.

### 2.4. Exclusion Criteria

This narrative review applied five exclusion criteria to ensure thematic precision and scientific rigor: (1) studies were dismissed if they lacked relevance to probiotics, prebiotics, synbiotics, or fermented dairy products, especially when not related to immune, or cardiovascular health; (2) low-quality research, such as non-peer-reviewed articles, poorly designed trials, or those with insufficient statistical grounding, was excluded; (3) outdated or redundant publications, including those predating 2000 or duplicating previously reviewed data, were filtered out; (4) language constraints led to the exclusion of studies not published in English or Spanish; and (5) articles that failed to align with the Boolean search strategy or omitted key terms like microRNAs, milk exosomes, or cardiometabolic outcomes were not considered.

### 2.5. Study Quality

This study adheres to the methodological quality standards for a narrative review as assessed by the SANRA criteria [11]. The relevance and importance of the topic, linking dairy-derived bioactives to gut microbiota and cardiometabolic, immune, and mental health, were clearly justified within the scientific perspective explained along the discourse of this study. In addition, the aims of this review were explicitly defined, focusing on precision nutrition and biologically active dairy components. A comprehensive literature search was conducted using relevant scientific databases of the field of knowledge (PubMed, Web of Science, and Google Scholar), applying a structured Boolean strategy across a defined publication window (1 January 2003 to 31 July 2025). In addition, referencing was thorough and has reflected a balanced integration of primary studies and regulatory sources. The level of evidence is critically appraised, favoring peer-reviewed, human-based research with mechanistic relevance. Furthermore, endpoint data, such as immune modulation, microRNA signaling, and cardiometabolic outcomes were consistently presented and contextualized.

## 3. Results

### 3.1. Nutritional and Functional Composition of Milk and Fermented Dairy Products

#### 3.1.1. Macronutrients and Micronutrients

Animal-derived milk, particularly from bovine sources, is one of the most widely consumed and nutritionally complete food products globally. Its widespread acceptance is attributed not only to its accessibility and versatility but also to its dense composition of essential macronutrients and micronutrients. The nutritional and bioactive composition of milk and milk-derived products is modulated by a complex interplay of intrinsic factors including breed, genetics, and lactational stage, as well as extrinsic factors such as animal dietary regimens, agricultural management protocols and seasonal environmental conditions [14,15]. In its natural unprocessed form, bovine milk primarily comprises water (~87%) with the remaining constituents including carbohydrates (4–5%), fats (3–4%), proteins (~3%), mineral elements (0.8%), and vitamins (0.1%), each contributing to its nutritional and functional properties [16].

##### Carbohydrates

Lactose represents the principal carbohydrate constituent in milk products and is uniquely characteristic of dairy-derived products. Structurally, it is a disaccharide formed by the linkage of glucose and galactose via a β-1,4 glycosidic bond. Upon enzymatic hydrolysis mediated by lactase in the small intestine, lactose is cleaved into its monosaccharide components, thereby facilitating efficient absorption and serving as a bioavailable energy substrate. In fermented dairy products, lactic acid bacteria metabolize substantial quantities of lactose into lactic acid and other byproducts, resulting in lower residual lactose content. This biochemical transformation enhances gastrointestinal tolerance, particularly among individuals exhibiting lactase deficiency, thereby making fermented dairy products more suitable for lactose intolerant populations [17,18]. In contrast to human milk, which contains approximately 12 g/L of oligosaccharides, bovine milk contains lower concentrations averaging around 1 g/L [19]. Nonetheless, over 200 structurally distinct oligosaccharide have been identified in bovine milk, several of which exhibit molecular similarities to their human milk counterparts. These bovine milk oligosaccharides (BMOs) exhibit prebiotic potential by selectively promoting the growth of beneficial gut microorganisms and acting as decoy receptors that inhibit the adhesion of pathogenic bacteria to the intestinal epithelial mucosa (Table 1) [20,21]. In this regard, BMOs such as 3′-sialyllactose and 6′-sialyllactose are structurally like human milk oligosaccharides and selectively enrich *Bifidobacterium* species [22]. In fact, symbiotic formulations combining BMOs with *B. animalis* subsp. *lactis* have shown a 100-fold increase in bifidobacteria in infant stool samples [23]. In addition, BMOs act as decoy receptors, preventing pathogen adhesion to the intestinal epithelial mucosa (Table 1).

##### Proteins

Milk is a rich dietary source of high biological value proteins, contributing approximately 32 g/L to human nutrition [26]. These proteins are traditionally classified into two main fractions: insoluble caseins, which account for approximately 80% of the total protein content, and the soluble whey proteins, comprising the remaining 20%. Caseins are particularly rich in essential amino acids such as histidine, methionine, and phenylalanine, whereas whey proteins are distinguished by their elevated concentrations of branched-chain amino acids, specifically leucine, isoleucine, and valine, which play a key role in regulating muscle protein synthesis and metabolic homeostasis [27].

Beyond their nutritional value, whey proteins encompass several bioactive components, including immunoglobulins, α-lactalbumin, β-lactoglobulins, lactoferrin, and casein phosphopeptides, exerting a wide variety of functional effects (Table 2). The proteolytic processing of milk proteins during gastrointestinal digestion and microbial fermentation facilitates the release of bioactive peptides which have demonstrated antibacterial, antiviral, antifungal, antioxidant, immunomodulatory, and antihypertensive properties, along with the capacity to enhance mineral bioavailability [28,29].

Lactoferrin, for instance, has been extensively studied for immunological and microbiological effects, including antimicrobial action, modulation of inflammation, and the promotion of mucosal immune equilibrium. It is known to enhance NK cells activity and regulate T-cell responses [30]. Likewise, milk-derived immunoglobulins contribute to passive immune protection by neutralizing microbial pathogens and reinforcing mucosal defense mechanisms [31].

**Table 2:** Protein composition of milk.

Proteins	Concentration (g/L)	Biological Function	References
Caseins			
α-casein	13	Antioxidant, mineral transport	[32]
β-casein	9.3	Antioxidant, antihypertensive, mineral transport	[32]
κ-casein	3.5	Mineral transport, inhibition of platelet aggregation	[32]
Total whey proteins			
β-lactoglobulin	7.5	Affinity for retinol and fatty acids, antioxidant	[33]
α-lactoalbumin	1.2	Calcium transport, immunomodulatory function, lactose production	[34]
Inmunoglobulin G1	9	Immune protection	[35]
Albumin	30	Transport	[33]
Lactoferrin	0.2	Antimicrobial, iron absorption, antihypertensive	[36]
Lactoperoxidase	0.03	Antimicrobial	[37]
Lisozime	0.0004	Antimicrobial, synergy with immunoglobulins and lactoferrin	[38]

##### Lipids

Lipids constitute the most variable macronutrient component within milk, with their concentration subject to intentional modulation through industrial processing techniques. Whole milk typically contains approximately 4% fat, whereas low-fat and skimmed variants contain about 1–2% and 0.5%, respectively [39].

The lipid fraction in milk represents a complex matrix, primarily composed of saturated fatty acids (60–70%), followed by monounsaturated (20–25%) and polyunsaturated fatty acids (3–5%) [40]. Among the polyunsaturated fatty acids, α-linolenic acid (omega-3) and linoleic acid (omega-6) are classified as essential fatty acids due to their endogenous non-synthesis and have been recognized for their anti-inflammatory, cardioprotective, and potential anticarcinogenic properties [41,42]. Moreover, bovine milk ranks among the richest natural dietary sources of CLA, a bioactive lipid implicated in the regulation of lipid metabolism, cognitive enhancement and risk mitigation of noncommunicable diseases [43].

Beyond its long-chain fatty acids constituents, such as palmitic, myristic, and stearic acids, bovine milk also contains appreciable quantities of short and medium chain fatty acids, including butyric, caproic, caprylic and capric acids. These fatty acids have demonstrated bioactivity encompassing antitumor, antiviral, and antimicrobial effects, and may contribute to the maintenance of intestinal barrier integrity and immunological homeostasis [44,45].

##### Vitamins and Minerals

Milk is a nutritionally balanced source of fat-soluble vitamins such as A, D, and E, and water-soluble B-complex vitamins including B2 (riboflavin), B12, and folate. While raw milk contains small amounts of vitamin C, this nutrient is highly sensitive to heat and is largely degraded during pasteurization, rendering its contribution nutritionally negligible in processed milk (Table 3) [46]. Among these, vitamin D is of biomedical relevance due to its multifactorial role in human health. Present at concentrations ranging from 5 to 40 IU/L in whole milk, vitamin D facilitates bone mineralization and contributes to calcium and phosphate homeostasis while also exerting immunomodulatory effects through the attenuation of inflammatory pathways, support of antiproliferative mechanisms, and enhancement in antimicrobial defenses [47].

Regarding its mineral content, milk provides substantial amounts of calcium (≈1200 mg/L), phosphorus (≈950 mg/L), and magnesium (≈120 mg/L) [49]. The high bioavailability of calcium from milk is attributed to the synergistic presence of lactose, casein phosphopeptides, and an optimally balanced calcium to phosphorus ratio, which collectively enhance intestinal absorption efficiency [50]. The food industry has significantly diversified its offerings to accommodate lactose-intolerant consumers. This includes lactose-free dairy products, processed by enzymatic hydrolysis of lactose into glucose and galactose, and fermented dairy (e.g., yogurt, kefir), which may contain lower lactose levels due to microbial breakdown during processing. However, traditional yogurt production often involves the addition of milk powder, which contains lactose, and the final lactose concentration can be similar to that of the original milk. Therefore, lactose reduction in yogurt is variable and depends on the specific manufacturing process. However, unhydrolyzed lactose may better support beneficial gut microbiota, indirectly enhancing calcium absorption, preserving milk and its derivatives as a functional balanced formula and a symbiotic functional food product with its dual role as a prebiotic, for its modulating gut microbiota dynamics, and a regulator of mineral transporter expression, for the improvement in calcium solubility and uptake. Lactose and galactooligosaccharides act as fermentable substrates for beneficial microbes like *Bifidobacterium* and *Lactobacillus*, promoting SCFAs production, which lower intestinal pH, stimulating calcium and magnesium receptors and improving gut barrier integrity [51,52]. Accordingly, dairy products constitute an effective dietary vehicle for maintaining bone health and preventing mineral deficiencies, particularly among at risk populations such as paediatric, adolescent, and elderly [53,54].

#### 3.1.2. Other Bioactive Compounds

In addition to providing essential nutrients and well-studied peptides, milk contains a variety of other bioactive compounds that contribute to its functional properties. These include signaling molecules, immune-modulatory agents, metabolic regulation, and components involved in host–microbiota interactions. Although typically present in small quantities, such compounds may exert substantial physiological effects, particularly when consumed regularly. Recent scientific finding underscores their potential roles in maintaining gut barrier integrity, modulating inflammatory responses, supporting neurodevelopment and promoting cardiometabolic health. The following sections offer a comprehensive overview of key emerging bioactive components in milk and dairy products, underscoring their relevance in the context of human health and precision nutrition.

##### Milk Fat Globule Membrane

The milk fat globule membrane (MFGM) is a trilayered phospholipid protein structure that encapsulates lipid droplets secreted by mammary epithelial cells, thereby stabilizing milk fat within the aqueous phase. Beyond its structural role, MFGM serves as a reservoir of bioactive compounds [55]. MFGM is composed of polar lipids such as phosphatidylcholine, sphingomyelin, and phosphatidylethanolamine as well as membrane bound proteins, including butyrophilin, xanthine oxidase, and mucin-1 [56]. These molecular components have been shown to exert a wide range of health promoting effects, notably anti-inflammatory, antimicrobial, and neurodevelopmental properties [57,58]. Emerging clinical evidence links MFGM-enriched dairy formulations with improved cognitive development in infancy, enhanced intestinal barrier function, and modulation of systemic immune responses [55,59]. Consequently, MFGM is increasingly acknowledged as a key functional element in dairy fat, with growing applications in both clinical nutrition and the innovation of functional food products with dairy formulations.

##### Milk-Derived Exosomes

Exosomes are nanoscale extracellular vesicles (30–150 nm) secreted by a variety of cell types, including mammary epithelial cells, and are naturally present in bovine milk. Structurally, they consist of a lipid bilayer that protects enclosed proteins, lipids, enzymes, and signaling molecules against enzymatic degradation and environmental stressors [60]. Proteomics analyses of bovine milk-derived exosomes (MDEs) have revealed an array of bioactive proteins, such as lactadherin and xanthine oxidase, involved in cell adhesion, antimicrobial defense, and modulation inflammatory responses [61]. Unlike other milk components, exosomes appear to exhibit stability under gastrointestinal conditions [62], although absorption and bioactivity have been demonstrated in in vitro and animal models, the exact amount of intact exosomes that reach the intestinal lumen and their bioavailability in humans remain uncertain. MDEs are emerging as bioactive vectors capable of mediating intercellular communication. Empirical evidence demonstrates their uptake by human intestinal and immune cells, where they engage in immune regulation and paracrine signaling [63]. These vesicles have been implicated in promoting gut microbiota composition, maintaining epithelial integrity and preserving homeostasis through paracrine signaling mechanisms [64,65,66]. Due to their natural origin, biocompatibility, structural stability, and capacity to transport diverse functional molecules, MDEs are emerging as promising candidates for oral delivery systems within food science, functional nutrition and therapeutic intervention paradigms [67].

MDEs have been incorporated into functional dairy products such as fortified yogurts, cheese, whey beverages and infant formulas, where they enhance gut immunity and nutrient absorption. In cosmeceuticals, they are used in milk-based skin creams and anti-aging supplements to promote collagen synthesis and reduce inflammation. Food processing technologies like ultracentrifugation, size exclusion chromatography, and electroporation are commonly used to isolate and load bioactive compounds into MDEs while preserving their integrity.

Clinically, MDEs have been explored in drug formulations like Exo@Dox-EPT1, a milk-exosome-based delivery system for oral squamous cell carcinoma, and XoLacta, a peptide-enriched milk exosome therapy for radiation protection. They have also shown promise in treating necrotizing enterocolitis, ulcerative colitis, and intestinal ischemia by restoring microbiota balance and reducing oxidative stress [68]. Recent studies have begun exploring MDEs in the context of food allergy, particularly bovine milk allergy. One study demonstrated that extracellular vesicles, including exosomes, derived from casein-induced allergic mice can modulate mast cells activation, suggesting a role in the pathophysiology and potential therapeutic targeting of cow milk allergy (CMPA) [69]. Another investigation showed that blocking exosome release reduced IgE-mediated hypersensitivity and Th2 cell activation in CMPA models [70], indicating that MDEs may influence immune responses relevant to food allergy.

However, the proposed roles of MDEs in immune regulation and paracrine signaling should be interpreted cautiously until validated in well-controlled clinical trials. Future research should focus on clarifying their bioavailability, functional stability, and dose–response effects in humans through multi-omics and controlled feeding studies.

##### Milk-Derived microRNAs

Beyond classical nutritional components, emerging insights from molecular biology have uncovered the presence of functional regulatory molecules in milk, particularly microRNAs (miRNAs). These molecules are short (~22 nucleotides), non-coding RNA sequences that modulate gene expression post-transcriptionally [71]. Although miRNAs are primarily synthesized endogenously by the host, dietary intake, especially from milk has been proposed as a significant contributor to the systemic miRNA pools, particularly during early development or under metabolic stress conditions [72].

In bovine milk, miRNAs are encapsulated within extracellular vesicles such as exosomes. These vesicles offer protection against enzymatic degradation, heat and acidic pH, thereby preserving the biological integrity of miRNAs [73]. These vesicles facilitate cellular uptake via endocytosis, enabling miRNAs to exert biological effects on distal tissues. It has been estimated that one liter of bovine milk contains approximately 140 mg of exosomes, and their ingestion has been associated with measurable increases in circulating levels of milk-derived miRNAs [72]. To date, over 400 unique miRNAs have been identified in bovine milk, many exhibiting high sequence homology to their human counterparts. These molecules have been implicated in a wide range of physiological processes, including embryogenesis, immune system modulation, haematopoiesis, and cardiometabolic regulation. For example, miR-26a has been linked to enhanced insulin sensitivity, attenuation of hepatic lipogenesis, and modulation of gluconeogenic pathways [74,75]. Although vesicle-encapsulated miRNAs may theoretically survive digestion and enter circulation, current human data remain inconsistent and largely indirect. Some studies have detected transient increases in circulating milk-derived miRNAs after consumption [76], whereas others found no detectable transfer across the intestinal barrier [77]. Therefore, while dietary miRNAs represent a promising avenue for interspecies communication, their physiological relevance in humans remains to be conclusively demonstrated.

#### 3.1.3. Fermented and Processed Dairy Products: Microbial and Nutritional Profiles

Fermented milk products are characterized by a high density of viable microorganisms, distinguishing them from conventionally processed milk, which exhibits a microbiologically inert profile. Ultra-High Temperature (UHT) milk is subjected to heat treatment at approximately 130 °C for one second, effectively eradicating both pathogenic (e.g., *Escherichia coli*, *Salmonella*, *Listeria*) and commensal bacterial populations. While this process ensures prolonged shelf life, the resultant product lacks viable bacteria. Pasteurization, in contrast, involves exposure to temperatures ranging from 72–90 °C for 12–15 s. This milder heat treatment substantially reduces microbial load while preserving a fraction of non-pathogenic bacteria. Notably, probiotic-enriched pasteurized milk and dairy products, such as cottage cow’s cheese, have shown to beneficially alter gut microbiota in murine models [78]. These changes are considered healthier than those induced by a standard diet, demonstrating the effectiveness of such products in delivering and sustaining probiotic bacteria in the gut [79,80]. Moreover, Zhang et al. demonstrated that bioactive miRNAs, which are often encapsulated in extracellular vesicles, are markedly degraded by UHT treatment, but largely preserved during pasteurization [81].

Among fermented dairy products, yogurt remains the most widely consumed type globally. It is produced through the controlled fermentation of milk by specific lactic acid bacteria, primarily *Streptococcus thermophilus* and *Lactobacillus delbrueckii* subsp. *bulgaricus* [82]. Beyond yogurt, a wide range of traditional and industrial fermented milk products have emerged worldwide, integrating diverse microbial strains from genera such as *Lactobacillus*, *Bifidobacterium*, and various yeasts such as *Saccharomyces cerevisae*. Table 4 shows representative microbial composition of several dairy products characterized by intricate, multispecies fermentation systems. This list is not exhaustive, and it may vary across different production systems and geographic regions. These microbial consortia interact synergistically, generating unique metabolic profiles that shape the organoleptic properties of the final product (e.g., flavor, texture and aroma). Moreover, these fermentative systems contribute to enhanced bioactivity, particularly through their capacity to modulate gut microbiota composition and functionality and conferring host health benefits [83].

Cheese constitutes another nutritionally and functionally important dairy derivative product, which is obtained through the enzymatic and microbial coagulation of milk, primarily via lactic acid bacteria. This biotransformation process results in an extensive array of cheese varieties with distinct textures, flavors, profiles and nutrient compositions. As water content is progressively reduced during manufacturing and maturation, the residual matrix becomes enriched in proteins, lipids, and minerals, including calcium, phosphorus, and magnesium. Sodium, commonly added during the ripening stage to enhance flavors, ensure microbial stability and preservation contributes to the final mineral profile of cheese [84].

**Table 4:** Different fermented products derived from milk.

Product	Shelf Life (4 °C)	Fermentation Microorganism	Description	References
Cheese	months	*Lactococcus*, *Lactobacillus*, *Streptococcus*, *Propionibacter*	Fermented solid food product obtained from curdled milk of ruminant animals	[85,86]
Sour cream	28 days	*Lactococcus lactis* subsp. *lactis*	Fermented cream to which salt is added. It has a smooth texture and a tangy flavor	[87]
Crème Fraîche	10 days	*Lactobacillus*, *Leuconostoc*, *Pediococcus*, *Lactococcus*, *Streptococcus*	Fermented cream originating from France, with a higher fat content than sour cream	[88]
Filmjölk	14 days	*Lactococcus lactis*, *Leuconostoc*	Fermented dairy product of Scandinavian origin, produced from bovine milk	[89]
Yogurt	40 days	*Streptococcus thermophilus*, *Lactobacillus delbrueckii* subsp. *bulgaricum*	Fermented milk produced using thermophilic microorganisms	[82]
Kéfir	14 days	*Lactobacillus acidophilus*, *Saccharomyces cerevisae*, *Lactobacillus kefiranofaciens*, *Lactococcus lactis*, *Leuconostoc* spp., *Kluyveromyces marxianus*, *Kazachstania* spp., *Bifidobacterium bifidum*	Fermented dairy beverage, similar in consistency to liquid yogurt, traditionally originating from the Caucasian region	[90,91,92]
Koumiss	14 days	*Lactobacillus acidophilus*, *Kluyveromyces marxianus*, *Saccharomyces cerevisae*	Fermented dairy product of Asian origin, traditionally produced from mare’s milk	[93,94]
Viili	14 days	*Lactococcus lactis*, *Leuconostoc mesenteroides*, *Geotrichum candidum*	Fermented milk product of Scandinavian origin, produced through surface mold fermentation	[89]
Buttermilk	10 days	*Lactococcus lactis*, *Leuconostoc mesenteroides*	Fermented dairy product obtained from pasteurized milk using mesophilic lactic acid bacteria, characterized by a lower viscosity than cream and a slightly acidic flavor	[95]
Acidophilus milk	14 days	*Lactobacillus acidophilus*	Fermented milk product inoculated with *Lactobacillus acidophilus*, a probiotic bacterium commonly used for its health-promoting properties	[96]

### 3.2. Dairy-Gut Microbiota Interactions: Consequences for Immune Regulation and Dietary Sensitivities

In recent years, a deep paradigm shift has been transforming our understanding of human physiology and disease, driven by the expanding recognition of the gut microbiota as a fundamental modulator of health [97]. The term “human microbiota” refers to the entire community of microorganisms inhabiting the human body, including bacteria, viruses, fungi, archaea, and protozoa. On the other hand, “human microbiome” encompasses not only these microorganisms but also their derived metabolites and dynamic interactions with the host, creating a vast and complex ecosystem. Although microbial niches are distributed across multiple tissues, the gastrointestinal tract, particularly the colon, harbors the highest density and diversity of microorganisms, comprising approximately 10^14^ microbial cells spanning nearly 1000 different species [98]. The main phyla in the gut microbiota are Firmicutes (Gram-positive) and Bacteroidota (Gram-negative), followed by Actinobacteria, Proteobacteria, and Verrucomicrobiota [99]. This complex microbial ecosystem has coevolved with its host over millions of years, establishing a dynamic symbiotic relationship that exerts wide-ranging physiological implications [100]. Once regarded as passive commensals, gut microbiota is now recognized as an active modulator of nutrient metabolism, immune system function, neurodevelopment, and maintenance of epithelial barrier integrity [101]. Evidence from germ-free/gnotobiotic animal models have been instrumental in elucidating the essential roles of the microbiota. These models, devoid of intestinal microorganisms, exhibit marked deficiencies in organ development, immune system maturation, metabolic homeostasis and behavioral responses such as mood alterations, pain perception, and influenced cognition, highlighting the gut microbiota as a key metabolic and neuroimmune organ [102].

The gut microbiota in healthy individuals exhibit a relatively stable composition over time. However, its structural dynamics are susceptible to modulation by a range of endogenous and environmental factors. These factors include mode of delivery, breastfeeding history, antibiotic exposure, and, most significantly, long-term dietary patterns [103,104]. Among these, diet has emerged as a principal driver of microbial variability with estimates suggesting up to 57% of interindividual variability in microbiota composition across the human lifespan [105].

Specific gut microbiota profiles may significantly influence lactose intolerance and milk protein allergies through distinct mechanisms. In lactose intolerance, bacteria such as *Bifidobacterium* and *Lactobacillus*, known lactose-fermenters, can metabolize residual lactose in the colon, reducing symptoms like bloating and diarrhea. This is particularly relevant for individuals with lactase non-persistence, where undigested lactose reaches the colon and is fermented into SCFAs and gases, most commonly hydrogen and methane. Interestingly, increased *Bifidobacterium* abundance has been associated with both symptom mitigation and, paradoxically, symptom exacerbation depending on host genotype and dairy intake [106]. The carbohydrate implicated here is lactose, and its microbial fermentation can either alleviate or exacerbate symptoms depending on microbial composition and host factors.

**Figure 2:**
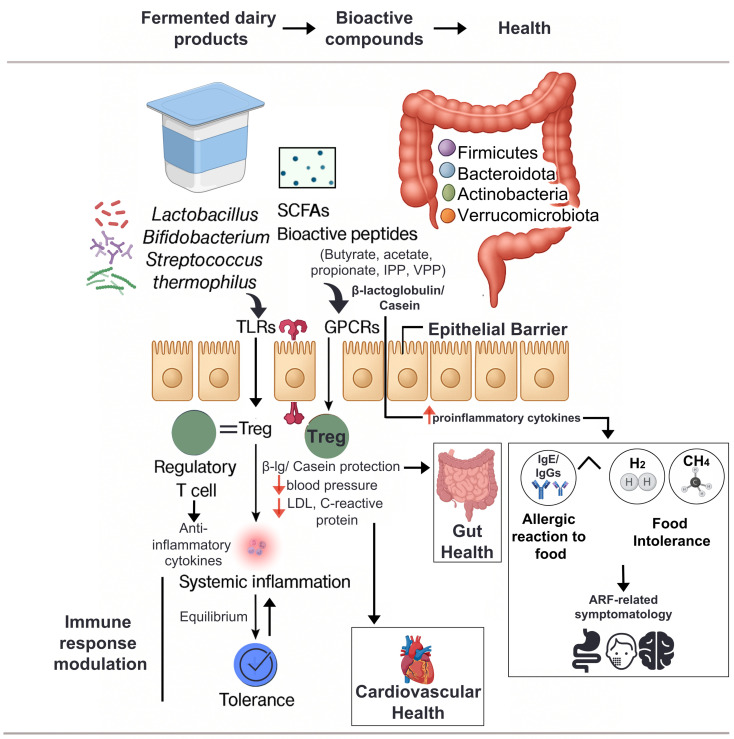
Molecular interactions between fermented dairy, gut microbiota, and immune modulation describing GPCR and TLR-dependent immunoregulatory pathways triggered by fermented dairy metabolites. ↑—increase; ↓—decrease. ARF, adverse reactions to food; CH_4_, methane; SCFAs, short-chain fatty acids; GPCRs, G protein-coupled receptors; H_2_, hydrogen; IgE, immunoglobulin E; IgG_4_, immunoglobulin G_4_; IPP, isoleucine-proline-proline; TLR, toll-like receptor; VPP, valine-proline-proline. Own elaboration using Microsoft Copilot software-based AI assistant GPT-4 (Microsoft Copilot for Microsoft 365, Microsoft Corporation, Azure and Open AI, Redmond, WA, USA; https://copilot.microsoft.com; accesed date: 22 August 2025), Venngage Multimedia and Design Software version Beta 2025 (Venngage Inc., Toronto, ON, Canada) and BioRender Software (BioRender Inc., Toronto, ON, Canada; https://www.biorender.com; accessed date: 16 October 2025). Disclosure: the schematic representations of cellular structures were generated based on descriptive prompts formulated by the authors in the mentioned software. These prompts were informed by foundational concepts drawn from Lehninger Principles of Biochemistry (Nelson & Cox, 5th ed.) [107] and Cell Biology by Gerald Karp (6th ed., 2010) [108], which served as primary references for cellular morphology and biochemical context. The remaining visual components, including representations of dairy products, directional arrows, icons, and layout elements, were constructed using standard forms and shapes available within Microsoft design tools, and were assembled to support the conceptual flow and clarity of the illustration.

Dairy protein allergies, especially CMPA, involve immune responses to proteins like β-lactoglobulin (a whey protein) and casein. Gut microbiota composition plays a role in modulating immune tolerance. Reduced diversity and depletion of beneficial taxa such as *Bifidobacterium* and certain *Clostridium* have been linked to persistent CMPA. These bacteria can promote regulatory T cell (Treg) differentiation and produce SCFAs like butyrate, which enhance gut barrier integrity and suppress inflammatory responses [106]. Hypoallergenic formulas enriched with prebiotics and probiotics have shown promise in restoring microbial balance and promoting tolerance acquisition (Figure 2).

#### 3.2.1. Gut Microbiota-Mediated Effects of Dairy Consumption

Dairy products are increasingly regarded as functional foods due to their bioactive components capable of modulating the gut microbiota composition. Multiple studies have shown that milk and its fermented derivatives promote the growth of *Bifidobacterium* and *Lactobacillus* species, commensal bacteria associated with anti-inflammatory responses, maintenance of epithelial barrier integrity, and elevated production of SCFAs [109,110,111,112,113].

A well-documented example is the colonic fermentation of lactulose, a lactose isomer, by *Bifidobacterium* and *Lactobacillus* leading to SCFA synthesis, acidification of the intestinal environment, inhibition of pathogenic bacteria, and preservation of mucosal integrity. These effects collectively support lactulose dual role as a prebiotic agent and a compound with antitumor potential [114,115]. Interestingly, in lactose-intolerant individuals, moderate dairy consumption may ameliorate gastrointestinal symptoms by promoting the growth of lactic acid bacteria capable of metabolizing residual lactose. Paradoxically, complete dairy exclusion may disrupt microbial adaptation, potentially exacerbating clinical manifestations [116,117].

Emerging evidence also implicates gut microbiota composition influencing immune responses to dietary antigens such as milk proteins. In cases of CMPA, specific microbiota profiles have been associated with differential gene expression in ileal epithelial cells, influencing the establishment or restoration of immune tolerance [118,119]. Collectively, these findings underscore the gut microbiota critical role in mediating host–diet interactions and support the development of personalized nutrition strategies employing prebiotic, probiotic, and symbiotic-based approaches to optimize dairy tolerance and gut health.

#### 3.2.2. Dairy-Gut Microbiota, and Immunonutrition: A Tripartite Interaction

In recent years, the role of nutrition in shaping immune function has gained growing attention, giving rise to the concept of immunonutrition as a field of study. This field explores how specific dietary components influence immune responses, both at the mucosal and systemic levels. Dairy products, particularly bovine milk and its fermented derivatives, are emerging as immunomodulatory agents due to their unique combination of bioactive compounds and their capacity to modulate the gut microbiota [78].

The rising prevalence of ARF, specifically CMPA and lactose intolerance across paediatric and adult populations has prompted a revaluation of underlying etiologies. While genetic predisposition and immune dysregulation are known contributors, recent evidence implicates the gut microbiota as a critical mediator in the initiation, persistence, and resolution of these ARF [120,121]. Microbial communities interact with gut-associated lymphoid tissue (GALT), modulating antigen presentation, cytokine secretion profiles, and epithelial barrier function. Milk-derived immunoglobulins, lactoferrin, MFGM and bioactive peptides collectively support these immunological processes, often acting synergistically with microbial metabolites and host immune cells [122].

Fermented dairy products such as yogurt and kefir promote the proliferation of beneficial genera including *Bifidobacterium* and *Lactobacillus*, which enhance SCFA production, reduce intestinal inflammation, and support epithelial integrity [109,110,111,112,113]. These microbial metabolites, particularly butyrate, have been shown to regulate dendritic cell function, T-reg cell expansion, and overall immune tolerance [123,124]. Such interactions underscore the central role of the dairy–gut microbiota axis in immune regulation, dietary sensitivities, and systemic inflammation. Emerging evidence suggests that disruptions in this axis may extend beyond allergic pathologies, potentially influencing cardiovascular risk profiles. Microbiota-derived metabolites generated through dairy fermentation have been associated with improved lipid metabolism and reduced systemic inflammation, linking dietary interventions with cardioprotective outcomes [5].

Alterations in gut microbiota composition have been implicated in ARF such as CMPA. Studies suggest that dysbiosis may impair oral tolerance mechanisms and contribute to aberrant Th2-mediated responses in individuals with positive CMPA [125,126]. Conversely, specific microbial profiles are associated with the resolution of CMPA and the restoration of immune tolerance [127]. In this regard, several studies have shown that children who outgrow CMPA exhibit specific shifts in gut microbiota composition. Following the acquisition of tolerance and the reintroduction of dairy products, a significant increase in lactic acid bacteria has been observed, along with a reduction in protein fermentation-derived metabolites. These changes suggest a partial restoration of microbial balance and a more immunologically favorable intestinal environment [128]. Therefore, higher relative abundances of *Bifidobacterium* and *Akkermansia muciniphila* have been associated with improved growth parameters and a healthier microbial profile. In contrast, children with active CMPA tend to exhibit increased proportions of *Bacteroides* and *Clostridium* species, which may reflect a pro-inflammatory or dysbiotic state [129]. These dysbiotic profiles may impair oral tolerance by disrupting antigen processing and mucosal immune signaling.

Collectively, these findings highlight the interplay between dairy-derived nutrients, intestinal microbes, and host immunity. Understanding this tripartite relationship may inform the development of microbiota targeted nutritional strategies for supporting immune health and managing food-related immune disorders. Modulating the gut microbiota through controlled exposure, prebiotics, or probiotic supplementation may offer new therapeutic strategies for promoting tolerance and ameliorating symptoms in dairy-related allergies and intolerances [130,131].

### 3.3. Dairy Intake and Cardiometabolic Health

Over the past decade, growing interest has been directed toward understanding the relationship between dairy product consumption and cardiometabolic health outcomes. Although some studies have reported neutral associations, growing epidemiological evidence suggests an inverse correlation between dairy intake and the risk of developing cardiovascular diseases (CVD), metabolic syndrome or type 2 diabetes mellitus [132,133].

In paediatric and adolescent populations, a systematic review [134] and the Healthy Lifestyle in Europe by Nutrition in Adolescence (HELENA) cross-sectional study [135] have reported that higher dairy consumption is associated with reduced adiposity markers, and a more favorable cardiometabolic profile. These benefits are thought to be mediated not only by the high nutrient density of dairy but also by the modulation of inflammatory markers, lipid metabolism, and glycaemic control [136,137]. In this regard, fermented dairy products such as yogurt and kefir might offer additional advantages due to their probiotic content, which can influence gut microbiota composition and promote metabolic and vascular homeostasis [138].

#### 3.3.1. Dairy Fat and Lipid Metabolism

CVDs remain the leading cause of mortality in industrialized nations, with elevated plasma cholesterol and saturated fatty acid levels recognized as key modifiable risk factors. Historically, this association has raised concerns about the potential atherogenic effects of dairy products, particularly whole fat varieties. However, current clinical evidence challenges this long-standing assumption. In this sense, a two-year cluster-randomized controlled trial conducted in 60 aged care homes in Australia evaluated the effects of increasing dairy intake to recommended levels using milk, yoghurt, and cheese on serum lipid profiles in older adults. The intervention group (n = 159) increased their intake to 3.5 servings/day, while the control group (n = 86) maintained approximately two servings/day. After 12 months, no significant differences were observed between groups in total cholesterol, LDL-C, HDL-C, triglycerides, or ApoB/ApoA-1 ratios, suggesting that increasing dairy intake in this population does not adversely affect lipid metabolism [139].

Furthermore, prospective cohort studies comparing full-fat and low-fat dairy products have not consistently supported universal recommendations favoring low-fat options [140,141]. Notably, interventional trials indicate that semihard bovine-cheese-based diet results in lower LDL-C and HDL-C concentrations compared to butter, despite both products having similar saturated fat content. In contrast, comparisons between cheese and milk have revealed no significant differences in lipid profiles [142]. These findings suggest that the impact of dairy fat on lipid metabolism may depend more on the food matrix than on saturated fat content alone, underscoring the need for further well-controlled trials to clarify metabolic effects.

#### 3.3.2. Dairy Intake and Hypertension

Hypertension is a primary modifiable risk factor for CVD and a major contributor to global morbidity and mortality. Among dietary strategies aimed at blood pressure control, the Dietary Approaches to Stop Hypertension (DASH) study was among the first to highlight the potential benefits of low-fat dairy consumption in reducing systolic and diastolic blood pressure levels [143]. The antihypertensive effect of dairy products has been attributed, in part, to their high content of calcium, magnesium, and potassium, minerals that synergistically support vascular homeostasis by modulating sodium balance, promoting vasodilation, and inhibiting angiotensin-converting enzyme (ACE) activity [144].

Moreover, some bioactive peptides, including casokinins and lactotripeptides, released during gastrointestinal digestion of casein and whey proteins, exhibit ACE-inhibitory properties and have been associated with slight clinically relevant reductions in blood pressure [145,146,147]. Furthermore, a generation of ACE-inhibitory peptides with antihypertensive properties derived from fermented milk products has been reported [148] (Table 5). Epidemiological evidence supports these mechanistic findings, for example, the Rotterdam Study, a large population-based cohort, suggest that higher dairy intake may be associated with a reduced risk of developing hypertension overtime, with some analysis indicating a potential reduction of around 20% in incidence (interpretation based on observation with stronger effect at 2 years, attenuated at 6 years, *p* < 0.08 both) [149].

Among the most studied casein-derived peptides with potential antihypertensive effects are Val-Pro-Pro (VPP) and Ile-Pro-Pro (IPP), which exert their activity primarily through ACE inhibition [150,151] These bioactive peptides VPP and IPP have demonstrated antihypertensive properties through ACE-inhibitory mechanisms in both preclinical animal models and human clinical trials. In addition to their vascular effects, these peptides have been associated with anti-inflammatory activity, suggesting their potential utility as nutritional adjuncts in the multifactorial management of metabolic syndrome [152].

In a randomized, double-blind, placebo-controlled trial by De Leeuw, P. W., et al., dairy peptides intake, specifically VPP and IPP, for 8 weeks resulted in a significant reduction in systolic blood pressure (SBP) by ~3.5–4.5 mmHg and diastolic blood pressure (DBP) by ~2.5–3.5 mmHg in mildly hypertensive subjects (*p* < 0.05) [153]. Similarly, a study by Seppo et al. reported that consumption of Lactobacillus helveticus-fermented milk containing VPP and IPP peptides led to a mean SBP reduction of 6.7 mmHg and DBP reduction of 3.6 mmHg over a 10 to 21-week period, with statistically significant differences compared to placebo (*p* < 0.06) [154]. Beyond αs1- and β-casein, κ-casein-derived peptides have also been implicated in vascular modulation, although their clinical impact remains less consistent. The physiological relevance of these peptides depends on multiple factors, including bioavailability, gastrointestinal stability, and individual metabolic response, which continue to be areas of active investigation. While mechanistic evidence supports their ACE-inhibitory potential, the magnitude of blood pressure reduction in clinical settings is generally modest and variable, underscoring the need for cautious interpretation and further research.

Together, these data support the inclusion of dairy products, particularly those rich in minerals and bioactive peptides, as part of a comprehensive dietary approach to blood pressure management.

#### 3.3.3. Effects of Dairy Consumption on Systemic Inflammation

While acute inflammation is a necessary component of host defense, chronic low-grade inflammation is increasingly recognized as a central mechanism in the pathogenesis of CVDs. In this context, several cross-sectional studies have reported an inverse association between dairy intake and circulating levels of pro-inflammatory biomarkers, including C-reactive protein (CRP) and IL-6 [155,156]. For example, individuals consuming more than 14 servings of dairy per week exhibited significantly lower levels of these inflammatory markers compared to those consuming fewer than eight servings [157]. The anti-inflammatory effects of dairy appear to be particularly relevant in individuals with metabolic risk factors. In a pilot study conducted by Nestel et al., only fermented dairy products, among various types tested, led to a significant reduction in IL-6 concentrations in overweight participants [158]. These findings suggest that fermentation-derived components or live microbial cultures may play a key role in modulating inflammatory pathways.

As previously mentioned, emerging molecular evidence points to the contribution of milk derived miRNAs, particularly those encapsulated in exosomes, as potential mediators of the anti-inflammatory response. For instance, miR-124a has been shown to suppress the expression of monocyte chemoattractant protein-1 (MCP-1), a critical chemokine for inflammatory cells recruitment [159]. Moreover, other miRNAs abundant in milk, such as miR-223, miR-199a, miR-155, miR-146a/b, miR-145 and miR-16 are involved in the regulation of toll-like receptor (TLR) and IL-1 signaling pathways, contributing to the attenuation of systemic inflammation [160]. Collectively, these data support the potential of dairy products, particularly fermented varieties enriched in bioactive molecules and miRNAs, to mitigate chronic inflammation and thereby reduce cardiometabolic risk.

### 3.4. Gut Microbiome-Heart Axis and Its Interaction with Cardiometabolic Health

Historically viewed as independent systems, the gut and cardiovascular system are now recognized as functionally interconnected via a complex network of metabolic, immunological, and neurohumoral pathways. Increasing evidence from both experimental and clinical studies supports a bidirectional relationship in which alterations in gut microbiota composition can significantly influence cardiometabolic outcomes. In animal models of myocardial ischemia/reperfusion injury, antibiotic-induced modulation of the gut microbiota has been shown to reduce infarct size and improve post-ischemic cardiac function, suggesting a causal role of microbial composition in cardiac resilience [161].

Several mechanisms have been proposed to explain the gut–heart axis. First, loss of microbial diversity and depletion of SCFA-producing bacteria impair intestinal barrier function, facilitating bacterial translocation and triggering both local and systemic inflammation [34]. Additionally, microbial metabolites exert systemic effects, for example, SCFAs (e.g., acetate, propionate, butyrate) modulate blood pressure, glucose metabolism, and immune function [162,163]; uremic toxins such as indoxyl sulphate and *p*-cresol promote vascular inflammation and endothelial dysfunction [164]; phenylacetyl glutamine (PAGln) is associated with CVD and death in humans promoting platelet invasiveness and thrombosis through adrenergic receptors [165]; and microbial modulation of gut hormones like ghrelin, GLP-1, and peptide YY influences appetite regulation and energy homeostasis [166].

Probably, trimethylamine N-oxide (TMAO) is the best-known bacterial metabolite. It is a hepatic oxidation product of trimethylamine generated from microbial metabolism of dietary L-carnitine and phosphatidylcholine (sourced of in red meat, eggs, and some dairy). It has been strongly associated independently with increased atherosclerosis risk, arterial stiffness, and cardiovascular events, even in individuals without conventional risk factors [167]. In this regard, fermented dairy products have shown to elicit a lower postprandial response of TMAO [168].

Regarding cardiac disease, a longitudinal study demonstrated that clinical recovery from a first episode of heart failure is accompanied by favorable shifts in gut microbiota composition, characterized by an increase in SCFA-producing genera such as *Bifidobacterium*. Notably, elevated faecal butyrate concentrations were positively associated with improved endothelial function and inversely correlated with levels of NT-proBNP and systemic inflammatory markers. Moreover, faecal supernatants from recovered individuals induced significantly lower nuclear factor kappa B (NF-κB) activation in HT29 colonic epithelial cells, suggesting a diminished intestinal proinflammatory capacity [169]. In reference to atherosclerosis disease, a recent and elegant study, show that imidazole propionate (ImP), another gut microbiota–derived metabolite, was identified as a novel driver of atherosclerosis in both mice and humans. Elevated plasma ImP levels correlated with subclinical and metabolically active atherosclerosis independently of circulating cholesterol levels [170].

Together, these findings underscore the gut microbiota as a potential therapeutic target for the prevention and management of cardiometabolic diseases. Interventions that preserve microbial diversity and promote beneficial taxa may offer cardioprotective benefits beyond traditional risk factor control.

### 3.5. Physical Activity and Lifestyle Modulation in Dairy Gut-Microbiome Interactions

Recent evidence underscores the bidirectional relationship between physical activity and gut microbiota composition, with lifestyle factors playing a pivotal role in shaping microbial diversity and function. Moderate-intensity exercise has been shown to enhance microbial richness and increase the abundance of SCFA-producing bacteria, such as *Faecalibacterium* and *Roseburia*, which contribute to improved gut barrier integrity and systemic immune regulation [171]. On the contrary, excessive or high-intensity training may induce gut dysbiosis and increase intestinal permeability, potentially leading to systemic inflammation [172]. Lifestyle components such as sleep quality, circadian rhythm, and stress levels further modulate microbial dynamics, suggesting that integrative lifestyle interventions with multifactorial approaches may synergistically support gut health [173].

Recent studies have explored the impact of fermented dairy products on athletic performance and recovery. A randomized crossover trial investigated the effects of fermented milk on glucose metabolism and muscle soreness in healthy young men following resistance exercise. Participants who consumed fermented milk before and after training showed improved glucose utilization, reduced muscle soreness, and enhanced antioxidant capacity compared to placebo [174]. Additionally, a review highlighted that probiotic supplementation, often delivered via fermented dairy may improve sports performance by modulating immune responses, reducing gastrointestinal symptoms, and enhancing nutrient bioavailability during high-intensity exercise. These findings suggest that fermented dairy products may support both metabolic resilience and physical recovery in athletic populations [175]. Another study analyzed metagenomic data from athletes and sedentary individuals, revealing that athletes tend to harbor gut microbes capable of producing SCFAs and beneficial compounds like vitamin B12 and amino acid derivatives. It supports the idea that diet, including probiotic-rich foods, can shape a microbiome that may potentially enhance physical performance [176].

**Figure 3:**
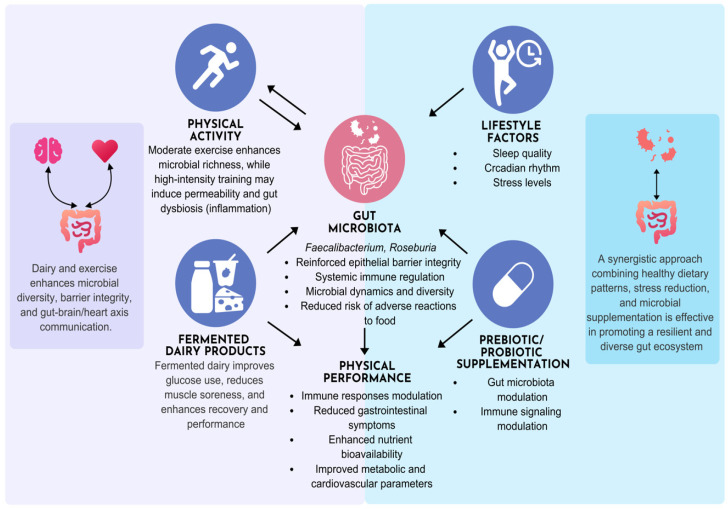
Physical activity, lifestyle & dairy gut-microbiome interactions. Own elaboration using Venngage Multimedia and Design Software (Venngage Inc., Toronto, ON, Canada).

Physically active individuals consuming fermented dairy products, either through diet or through targeted prebiotic/probiotic supplementation, may experience multifactorial health benefits via modulation of the gut microbiota. Gao et al. reviewed mechanistic and clinical data showing that fermented dairy foods such as yogurt, kefir, and cultured milk beverages enhance microbial diversity, reinforce epithelial barrier integrity, and modulate immune signaling, contributing to improved metabolic and cardiovascular parameters [177]. Abd El-Salam et al. further demonstrated that probiotic-rich fermented milk could endure gastrointestinal transit and beneficially alter gut microbiota composition, potentially reducing the risk of ARF, specifically food allergies and intolerances, through immune regulation and microbial competition [178]. These microbiota shifts are also linked to enhanced gut–brain and gut–heart axis communication, supporting neuroimmune resilience and cardiovascular health in active populations. Collectively, these findings highlight the potential of fermented dairy as a functional dietary strategy to optimize performance and systemic health in athletes and physically active individuals (Figure 3).

### 3.6. Metagenomics and Personalized Nutrition Approaches

The human gut microbiota exhibits remarkable genetic complexity, encoding over three million microbial genes, vastly surpassing the approximately 25,000–30,000 genes in the human genome. Collectively referred to as the metagenome, this microbial gene pool confers metabolic and regulatory capabilities that far exceed those of the host, yet many of its components remain functionally uncharacterized [179].

The advent of next-generation sequencing (NGS) technologies has transformed both microbiome research and clinical diagnostics. By enabling high-throughput analysis of microbial DNA directly from biological samples, without the need for culturing, NGS has facilitated the emergence of metagenomics, allowing for comprehensive taxonomic and functional profiling of microbial communities and their relevance to host physiology and disease [180]. Metagenomic studies have revealed that the gut microbiota contributes substantially to interindividual variability in numerous health-related traits, including metabolic response, immune modulation, and drug metabolism [181,182]. Unlike the relatively static human genome, the microbiome is dynamic and highly responsive to environmental inputs, particularly diet [183]. This plasticity makes it a compelling target for interventions using prebiotics, probiotics, symbiotics, and even faecal microbiota transplantation (FMT) [184].

Within this framework, the long-standing and global consumption of dairy products suggests a possible coadaptation between human biology and gut microbiota to this nutrient rich food [185]. As previously demonstrated in other foods such as bread [186], interindividual differences in microbiota composition may underlie the variability observed in metabolic, immunological, or cardiovascular responses to dairy intake [187]. Understanding these host–microbe–diet interactions is therefore essential for advancing precision nutrition, whereby dairy-based interventions can be tailored to individual microbiome profiles to maximize health benefits.

## 4. Discussion

The findings summarized in this review reinforce the concept that dairy products, particularly fermented varieties, represent more than basic nutritional sources. They act as bioactive matrices capable of modulating gut microbiota composition and influencing systemic physiological processes, including immune, ARF, physical performance and cardiometabolic regulation.

A recurring theme across multiple sections is the tripartite interaction between dairy-derived bioactive compounds, the gut microbiota, and host immunity. Lactic acid bacteria in fermented products such as yogurt and kefir consistently promote the proliferation of commensal genera like *Lactobacillus* and *Bifidobacterium*, which are known to enhance epithelial integrity, reduce intestinal inflammation, and contribute to SCFA production [188,189,190]. These effects may be especially important in the context of food hypersensitivities, such as CMPA, where microbial imbalances can modulate antigen presentation and immune tolerance [117].

Moreover, dairy-derived components such as milk exosomes, miRNAs, lactoferrin, and MFGM have emerged as key immunonutritional agents. Their potential roles in immune modulation and inflammatory signaling attenuation provide a plausible biological rationale, although these effects require further confirmation in well-designed human trials against low-grade inflammation and NCD [191,192]. In this regard, a recent systematic review and meta-analysis of 53 randomized controlled trials (n = 3215) evaluated the effects of milk protein supplementation, including whey and casein, on inflammatory biomarkers in adults. Although no significant changes were observed for CRP, TNF-α, leptin, or adiponectin, a statistically significant reduction in serum IL-6 levels was reported in the intervention group compared to controls (WMD: −0.25 pg/mL; 95% CI: −0.48 to −0.03; *p* = 0.026). This anti-inflammatory effect was more pronounced in trials lasting over 8 weeks and using low doses (<30 g/day), particularly among women and individuals with obesity. These findings suggest that milk proteins may exert modest but clinically relevant immunomodulatory effects, especially on IL-6, a key cytokine in chronic low-grade inflammation [158].

In parallel, the role of dairy products in food hypersensitivities such as cow’s milk protein allergy (CMPA) and lactose intolerance warrants careful consideration. While these conditions are often cited as reasons to restrict dairy intake, emerging evidence suggests that certain dairy matrices, particularly fermented products, may support immune tolerance and symptom mitigation. Fermentation processes can reduce the allergenic potential of milk proteins and enhance the bioavailability of immunomodulatory compounds, while also introducing beneficial microbial strains that contribute to gut barrier integrity and immune regulation. Moreover, a recent umbrella review of 41 meta-analyses encompassing 45 unique health outcomes highlighted that although milk consumption may slightly increase the risk of iron-deficiency anemia in infancy, the overall prevalence of CMPA remains relatively low (0.6–3.0%), and most individuals with lactose intolerance can tolerate up to 12–15 g of lactose (approximately one cup of milk) without symptoms [193]. These findings underscore the importance of distinguishing between different types and processing methods of dairy products, as well as considering individual tolerance thresholds. Rather than being uniformly contraindicated, dairy, especially fermented varieties, may offer therapeutic potential in managing certain food intolerances and allergies, particularly when integrated into microbiota-targeted dietary strategies.

From a cardiovascular point of view, dairy intake, particularly fermented products, has been associated with reduced blood pressure, favorable lipid profiles, and lower levels of inflammatory biomarkers, without consistent adverse effects from saturated fat content [194]. For example, a dose–response meta-analysis of 9 prospective cohort studies (n = 57,256; 15,367 hypertension cases; follow-up: 2–15 years) found that each 200 g/day increase in total dairy intake was associated with a 3% lower risk of hypertension (RR: 0.97; 95% CI: 0.95–0.99). Similar inverse associations were observed for low-fat dairy (RR: 0.96; 95% CI: 0.93–0.99) and milk (RR: 0.96; 95% CI: 0.94–0.98), with no significant heterogeneity across studies. These findings suggest a modest but consistent protective effect of dairy particularly low-fat varieties on blood pressure regulation [144]. Additionally, novel mechanistic pathways such as inhibition of ACE by bioactive peptides, and suppression of monocyte chemoattractant proteins by milk-derived miRNAs, further substantiate the cardioprotective potential of dairy constituents.

A particularly promising line of evidence concerns the gut–heart axis through metabolomic analyses, highlighting how microbial metabolites such as SCFAs, TMAO, PAGln or ImP can directly influence cardiovascular outcomes. The lower postprandial TMAO response observed after fermented dairy intake underscores the importance of food matrix effects and microbial mediation in determining metabolic impact. Likewise, the association between clinical recovery in heart failure and enrichment of SCFA-producing genera, especially butyrate, reinforces the therapeutic potential of microbiota-targeted dietary strategies.

Regarding physical performance, studies in athletes and physically active individuals have shown that the composition of the gut microbiota differs from that of sedentary individuals, with greater microbial diversity and a higher prevalence of health-promoting species [9]. Supplementation with probiotics and fermented dairy products may enhance recovery, exercise capacity, and overall health, although the effects vary depending on diet and baseline microbiome composition [195]. In this context, a systematic review by Alcantara et al. evaluated 11 studies involving 321 participants and found that cow’s milk intake, particularly in doses of 500 to 1000 mL, may attenuate losses in peak torque, improve countermovement jump and sprint performance, and reduce muscle soreness following resistance or high-intensity exercise. While the interventions ranged from acute protocols to a 12-week training program, the results were mixed, likely due to differences in study design, fitness level, and timing of ingestion. Although no pooled relative risks or confidence intervals were reported, this review supports a potential role for dairy-based strategies in enhancing physical recovery, which may be partially mediated by gut-muscle axis interactions [196].

Finally, advances in metagenomic technologies have illuminated the vast functional potential of the gut microbiome and its responsiveness to CVD. For example, in a spontaneously hypertensive heart failure rat model, sequencing of multiple hypervariable regions of the 16S rRNA gene has allowed for the identification of gut microbiota alterations that precede and accompany the transition to heart failure [197].

Given the interindividual variability in microbiome, precision nutrition approaches using fermented dairy as a modifiable dietary element may offer tailored solutions to optimize immune and cardiometabolic responses.

### 4.1. Research Gaps

Despite increasing evidence on the health benefits of fermented dairy products, most studies are observational and lack mechanistic insight. Further, randomized controlled trials integrating metagenomic, metabolomic, and immunologic data are needed to clarify causal relationships and individual variability in response.

### 4.2. Clinical Implications

Fermented dairy products may represent effective, accessible interventions to modulate the gut microbiota and support cardiometabolic and immune health. Their incorporation into clinical dietary guidelines should consider individual microbiome profiles to enhance therapeutic outcomes.

### 4.3. Limitations of This Review

As a narrative review, this work is subject to selection bias and does not provide quantitative synthesis. Additionally, heterogeneity across studies regarding product types, populations, and endpoints limits the generalizability of conclusions.

This narrative review synthesizes current evidence on the health effects of fermented dairy products, particularly their role in modulating the gut microbiome and influencing metabolic outcomes. However, several limitations must be acknowledged. First, the absence of a PRISMA-style flow diagram reflects the nature of this work as a narrative review, which does not require formal systematic mapping. Second, the heterogeneity of the studies included, ranging from randomized controlled trials (RCTs) to mechanistic and observational designs, limits the comparability of outcomes. Where possible, study types were distinguished and reported effect sizes and statistical indicators were analyzed, but variability in population characteristics, intervention protocols, and outcome measures may influence interpretation.

Additionally, the schematic elements in Figure 2 were generated using AI-assisted tools Microsoft Copilot software-based AI assistant (Microsoft Copilot for Microsoft 365, Microsoft Corporation, Azure and Open AI, Redmond, WA, USA), and Venngage Multimedia and Design Software (Venngage Inc., Toronto, ON, Canada) based on authors-directed prompts. Figure 2 should be interpreted as a conceptual aid based on the present manuscript rather than a definitive representation of a defined cellular process.

Finally, publication bias and confounding variables, such as dietary patterns, microbiome diversity, and host genetics, remain as future research considerations to evaluate the impact of fermented dairy products. These factors underscore the importance of personalized nutrition approaches and the need for future studies with standardized methodologies and clinically validated endpoints.

## 5. Conclusions and Future Directions

Despite the large body of research investigating the relationship between milk and dairy consumption, immunity, ARF, physical performance and cardiometabolic risk, findings remain inconsistent and often contradictory. This lack of consensus may be partially attributed to the traditional reductionist focus on isolated nutrients and metabolic biomarkers, overlooking food matrix and interindividual variability, particularly in gut microbiota composition. In this context, the integration of gut microbial profiling into nutritional evaluation emerges as a critical tool. As sequencing technologies become more accessible, it is conceivable that routine microbiome analysis will enable clinicians and researchers to assess individualized needs and predict responses to specific foods or therapeutic agents, paving the way toward precision nutrition and personalized medicine.

A more comprehensive understanding of the human microbial ecosystem could facilitate the design of precise interventions, including microbiota-guided dietary recommendations, tailored prebiotic, probiotic, or symbiotic strategies, and even precision-guided FMT. Unlike the human genome, the gut microbiome is highly plastic and modifiable, representing a promising and actionable target for therapeutic modulation.

In conclusion, bridging traditional nutrition science with microbiome research provides a more nuanced framework to understand the health effects of dairy products. Continued exploration of the diet–gut microbiota–host interactions will be essential for refining clinical dietary guidelines and for better understanding the potential contributions of fermented dairy products to cardiometabolic and immune health, particularly when combined with physical activity guided by a sports science professional. Further research, especially human clinical and multi-omics studies, is needed to confirm these effects.

## Figures and Tables

**Table 1:** Key probiotic bacteria stimulated by bovine milk oligosaccharides (BMOs).

Bacteria	Role & Relevance	References
*Bifidobacterium longum*	Promotes gut barrier integrity, reduces inflammation, and is commonly enriched by BMOs	[22]
*Bifidobacterium breve*	Supports immune modulation and is frequently found in infant gut microbiota	[23,24]
*Bifidobacterium bifidum*	Known for adhesion to intestinal mucosa and competitive exclusion of pathogens	[23]
*Bifidobacterium pseudocatenulatum*	Demonstrates persistence and metabolic activity when paired with BMOs like 2′-fucosyllactose	[24]
*Bifidobacterium animalis* subsp. *lactis* (*CNCM I-3446*)	Used in symbiotic formulas; shows strong bifidogenic effects when combined with BMOs	[23]
*Lactobacillus* spp. (e.g., *L. rhamnosus*, *L. casei*)	Though less dominant, some strains benefit from BMOs and contribute to gut health	[22]
*Parabacteroides distasonis*	Emerging evidence suggests BMOs may promote its growth, with anti-inflammatory potential	[25]

BMOs, bovine milk oligosaccharides.

**Table 3:** Vitamin concentrations in raw and UHT-processed milk (per 100 mL).

Vitamin	Raw Milk	UHT-Processed Milk
A (µg)	37	21
D (IU)	38	20
E (mg)	0.06	0.31
B1 (mg)	0.03	0.04
B2 (mg)	0.20	0.21
B3 (mg)	0.20	0.10
B6 (mg)	0.06	0.05
B12 (µg)	0.40	0.30
C (mg)	2.00	1.00
Folate (µg)	8.00	2.00
Pantothenic Acid (mg)	0.60	0.34

UHT, ultra-high-temperature treatment. Data according to Finglas et al. [48].

**Table 5:** ACE-inhibitory peptide sequences found in fermented bovine milk.

Casein	Peptide Fraction	Amino Acid Sequence	Inhibitory Concentration 50 (μM)
*α* *s1*	146–147	YP	720
	194–199	TTMPLW	51
	142–147	LAYFYP	65
	157–164	DAYPSGAW	98
*β*	114–115	YP	720
	74–76	IPP	5
	84–86	VPP	9
	193–198	YQEPVL	280
	108–113	EMPFPK	423
	177–183	AVPYPQR	274
	11–20	LVYPFPGPIH	89
	11–26	LVYPFPGPIPNSLPQN	71
*κ*	58–59	YP	720
	108–110	IPP	5

Adapted from Domínguez-González et al. [148].

## Data Availability

No new data were created or analyzed in this study.

## List of Abbreviations

ACE	Angiotensin-converting enzyme
ARF	Adverse reactions to food
BMOs	Bovine milk oligosaccharides
CLA	Conjugated linoleic acid
CMPA	Cow’s milk protein allergy
CRP	C-reactive protein
CVD	Cardiovascular disease
DASH	Dietary approaches to stop hypertension
FMT	Fecal Microbiota Transplantation
GABA	γ-aminobutyric acid
GALT	Gut-associated lymphoid tissue
HELENA	Healthy lifestyle in Europe by nutrition in adolescence
ImP	Imidazole Propionate
MCP-1	Monocyte Chemoattractant Protein-1
MFGM	Milk fat globule membrane
miRNAs	microRNAs
NGS	Next-generation sequencing
NF-κB	Nuclear factor kappa B
PAGln	Phenylacetyl glutamine
SANRA	Assessment of narrative review articles
SCFAs	Short-chain fatty acids
TLR	Toll-like receptor
TMAO	Trimethylamine N-oxide
UHT	Ultra-high temperature
